# Correction to “Association between continuous glucose monitoring metrics and diabetic retinopathy and albuminuria among Japanese individuals with type 1 diabetes”

**DOI:** 10.1111/jdi.70356

**Published:** 2026-06-10

Kondo Y, Kubota R, Suda R, Hasegawa Y, Oya J, Takagi S, Takaike H, Miura J, Nakagami T. Association between continuous glucose monitoring metrics and diabetic retinopathy and albuminuria among Japanese individuals with type 1 diabetes. *J Diabetes Investig*. 2026;17: 818–826. http://doi.org/10.1111/jdi.70287.

In the paragraph 2 of “Differences in CGM metrics, HbA1c, and clinical characteristics according to microvascular complications status” in the “RESULTS” section, the text “Individuals with albuminuria were significantly older, had a higher BMI, and had a longer duration of diabetes, higher HbA1c levels, a higher proportion of women, hypertension, and dyslipidemia, and lower eGFR levels than those without albuminuria.” was incorrect.

This should have read as follows: “Individuals with albuminuria were significantly older, had a higher BMI, and had a longer duration of diabetes, higher HbA1c levels, a lower proportion of women, higher proportions of hypertension and dyslipidemia, and lower eGFR levels than those without albuminuria.”

We apologize for this error.

